# Determinants of patient cycle time in primary healthcare: a large-scale retrospective study from Saudi Arabia

**DOI:** 10.3389/frhs.2026.1814326

**Published:** 2026-04-24

**Authors:** Malak A. Al Shammari, Leila A. Boubshait

**Affiliations:** Family and Community Medicine Department, College of Medicine, Imam Abdulrahman Bin Faisal University, Dammam, Saudi Arabia

**Keywords:** electronic health records, patient cycle time, patient flow, primary healthcare, quality improvement, Saudi Arabia, waiting time

## Abstract

**Background:**

Patient cycle time is a useful operational metric for assessing the clinical encounter pathway in primary healthcare settings. Prolonged cycle times may reflect delays within the consultation process and may affect patient experience and clinic flow.

**Objectives:**

This study aims to evaluate patient cycle time at a large Family and Community Medical Centre in the Eastern Province of Saudi Arabia and to identify the demographic, clinical, and operational factors associated with variations in cycle time.

**Methods:**

A retrospective analytical chart review was conducted using electronic health record data for all visits to FCMC over a 1-year period in 2025 (*n* = 52,611). Cycle time was defined as the duration from patient check-in to the closure of the physician encounter. Sociodemographic variables, visit characteristics, clinic type, seasonality, and diagnostic categories were analysed. Multivariable linear regression was performed to identify predictors of cycle time.

**Results:**

The median cycle time was 20 min (interquartile range: 20–21). Longer cycle times were significantly associated with visits to urgent care and chronic disease clinics, Saudi nationality, autumn visits, and acute diagnoses, including upper respiratory, gastrointestinal, musculoskeletal, neurological, and dermatological conditions. Shorter cycle times were observed for preventive care and diabetes-related visits. Increasing age was independently associated with shorter cycle time, while sex was not a significant predictor.

**Conclusion:**

Cycle time at this primary healthcare centre was relatively short compared with national and international benchmarks. However, it varied by clinic type, diagnosis, season, and patient characteristics. Targeted operational interventions focusing on urgent care services, seasonal demand, and acute presentations may help optimize the clinical consultation pathway.

## Introduction

In rapidly evolving healthcare systems, continuous evaluation of service delivery is essential to improve care quality and enhance patient satisfaction. Patient satisfaction can be defined as a patient's reaction to several aspects of their service experience (1). Assessing patient satisfaction provides valuable insight into service performance and helps prioritize areas for improvement and resource allocation.

Overall patient satisfaction is influenced by several factors related to the healthcare setting, facility management, quality of care, physician and allied health staff, and geographical context. Although some determinants may be unique to a particular setting, most generally fall within these broad categories (2). One of the most frequently cited factors influencing patient satisfaction in outpatient clinic settings is waiting time (3).

Waiting time is defined as the time a patient spends waiting for a specific service, such as a procedure or physician consultation. Cycle time is a quality metric usually defined as the total elapsed time from the moment a patient enters the facility (check-in) to the moment of checkout (4).

Prolonged cycle time can lead to patient overload and an imbalance between the influx and outflux of patients at each station in the Outpatient Clinic system (OPC) system and primary healthcare (PHC) centres (5). Different factors have been identified in the literature that may influence cycle time, including seasonal variations in service utilization and the timing and booking of appointments from morning to late afternoon sessions. Different specialty appointments within the same setting can cause delays, uneven queues, and added pressure on clinic workflow (5, 6).

Reducing cycle time in a PHC centre not only improves patient satisfaction and overall service outcomes but also enhances the coordination and planning of services and resources (6). These benefits encourage healthcare facilities to invest in quality improvement initiatives aimed at reducing cycle time, improving planning, enhancing patient experience, lowering no-show rates, and promoting more efficient use of resources (7).

Despite growing interest in healthcare operational efficiency, most studies in primary healthcare settings have focused primarily on isolated components of the patient journey, such as waiting time or consultation duration. However, patient cycle time, defined as the elapsed time between patient check-in and completion of the clinical encounter, serves as an indicator of efficiency within the clinical consultation pathway and patient flow. Unlike waiting time alone, cycle time captures the overall interaction within the clinical consultation pathway and therefore offers a more comprehensive perspective on service performance (8). In Saudi Arabia and the wider region, published evidence on the determinants of patient cycle time in primary healthcare settings remains limited, particularly studies that utilize large-scale electronic health record data. Understanding patterns in cycle time and their determinants is essential for improving clinic workflow, optimizing resource allocation, and enhancing patient experience. Therefore, evaluating cycle time within a large academic primary healthcare centre provides valuable operational insights to inform quality improvement initiatives and support ongoing healthcare transformation efforts under Saudi Vision 2030.

The objective of this study was to evaluate patient cycle time at a large Family and Community Medical Centre (FCMC) in the Eastern Province of Saudi Arabia and to explore factors associated with its variation to improve service quality and overall patient satisfaction.

## Materials and methods

### Study setting and design

This study is a retrospective analytical chart review based on electronic health record for all healthcare visits conducted over a 1-year period at the FCMC, Imam Abdulrahman Bin Faisal University. FCMC serves as the primary healthcare centre of the newly established Imam Abdulrahman Bin Faisal Academic Medical City, located in Khobar, Eastern Province, Saudi Arabia. The centre serves a population of more than 30,000 individuals, including students, faculty members, administrative staff, and their families, and also receives stable referrals from secondary and tertiary care services within the academic medical city. The study protocol was approved by the Institutional Review Board (IRB-2025-01-0905). All extracted data were fully anonymized prior to analysis. No identifiable patient information was accessed or included in the dataset, ensuring compliance with institutional and international ethical standards for retrospective studies.

FCMC provides a wide range of clinical services, including general family medicine clinics staffed by family medicine consultants, specialists, and training residents. In addition, several specialized clinics are available, such as geriatrics, women's health, metabolic, obesity, and diabetology clinics, all operated by family medicine physicians with relevant expertise. The centre also offers services from other medical, surgical, and allied health specialties, including ophthalmology, neurology, and physiotherapy. All health visits recorded in 2025 were initially retrieved from the electronic health record system (*n* = 54,264). Visits with invalid or incomplete timestamps required for cycle time calculation were excluded (*n* = 1,653; 3.05%). The final analytical dataset therefore comprised 52,611 visits with valid timestamp data.

Data were extracted from the electronic health record system (TrackCare). Patient arrival time was defined as the check-in and registration time at the front desk, while checkout time was defined as the timestamp at which the physician closed the encounter in the electronic health record and marked the patient as departed following completion of the consultation. In the study setting, additional services such as laboratory tests, imaging, procedures, or nursing interventions are typically recorded as separate service encounters and were therefore not included in the consultation cycle time. Visits with invalid timestamps were excluded. Collected variables included total time to departure (in minutes), age, gender, nationality, diagnosis, appointment time and type, department, and service.

### Data management and statistical analysis

Analyses commenced with data management. Using the month of the visit, a variable was created to group the months into seasons, with winter (December, January, February), spring (March, April, May), summer (June, July, August), and autumn (September, October, November). Clinic types were also grouped into the following categories: General Family Medicine (general family medicine, students clinics, geriatric medicine), Chronic Disease clinics (diabetes clinics, metabolic clinic, clinical nutrition, pulmonology, and cardiopulmonary clinics), Mental Health (mental health, adult and paediatric psychiatry, adult and paediatric psychology), Maternal, Child, and Women's Health (well baby clinic, child development, women's health), Preventive and Supportive Clinics (preventive medicine, health education, smoking cessation clinics), Procedure-Based clinics (dermatology, minor surgery, ophthalmology, retina clinic, radiology), and Rehabilitation and Allied Health (physiotherapy, musculoskeletal, neurology rehabilitation clinics). Diagnoses were categorized into 14 major groups: diabetes mellitus and glycaemic disorders (type 1 and type 2 diabetes, prediabetes), hypertension and cardiovascular conditions (essential hypertension, secondary hypertension and ischaemic heart disease), other metabolic and endocrine disorders (dyslipidaemia, thyroid disorders, obesity), mental health and behavioural disorders (depression, anxiety, dysthymia), upper respiratory tract infections (acute upper respiratory tract infections, pharyngitis, tonsillitis), lower respiratory tract infections (bronchitis, pneumonia, asthma exacerbations), gastrointestinal conditions (gastroenteritis, abdominal pain, gastritis), musculoskeletal and pain conditions (low back pain, joint pain, osteoarthritis), neurological conditions (headache, migraine, epilepsy), genitourinary (urinary tract infections, dysuria, prostate disorders), skin and dermatological conditions (dermatitis, eczema, acne vulgaris), women's health and pregnancy (confirmed pregnancy, abnormal uterine or vaginal bleeding), preventive care and follow-up (screenings, Hajj vaccinations), and general examinations or routine check-ups (laboratory examinations, general medical examinations, follow-up visits).

Cycle time was the outcome of the study and was found to be highly right-skewed. Therefore, a log transformation was applied to this variable. All categorical variables were described as frequencies and percentages, whereas age was reported as mean ± standard deviation, and cycle time was described as median and interquartile range. A multivariable linear regression was performed using the log-transformed cycle time variable as the outcome. The resulting coefficients, along with their 95% confidence intervals (CIs), were subsequently exponentiated, then were subtracted from 1, and expressed as percentages by multiplying the result by 100. All coefficients were presented in a forest plot to facilitate comparison and interpretation. Analysis was performed using Stata (9, 16).

## Results

### Sociodemographic and visit characteristics

The total number of visits during the study period was 52,611. Table 1 presents the sociodemographic characteristics of these visits. The mean age was 36.26 years (± 18.59). Approximately 60% of all visits were made by females, and Saudi nationals had the majority of visits (77.88%).

**Table 1:** Sociodemographic characteristics of all visits to the family and community medicine centre.

Characteristics	*N* (%) 52,611 (100.00)
Age (*µ*, SD)	36.26 (18.59)
Sex	
Male	21,295 (40.48)
Female	31,316 (59.52)
Nationality	
Saudi	40,975 (77.88)
Non-Saudi	11,636 (22.12)

Table 2 describes the visit characteristics. General family medicine clinics accounted for the highest proportion of visits (73.12%), followed by urgent care (19.77%). The most common diagnosis recorded for the visits was general examinations (64.72%), followed by diabetes and hypertension (12.63% and 5.93%, respectively). The least recorded diagnosis was lower respiratory tract infections (0.34%). The median cycle time for all visits was 20 min, with an interquartile range of 20–21 min. With regard to the season of visits, most visits occurred during autumn, followed by summer (30.76% and 26.0%, respectively), while the least proportion of visits was recorded in spring.

**Table 2:** Visit characteristics of all visits to the family and community medicine centre.

Characteristics	*N* (%) 52,611 (100.00)
Clinic type	
General family medicine	38,471 (73.12)
Urgent care	10,399 (19.77)
Maternal, child, and women's health	1,559 (02.96)
Chronic disease clinics	1,265 (02.40)
Procedure-based clinics	502 (00.95)
Mental health services	215 (00.41)
Preventive and supportive services	188 (00.36)
Rehabilitation and allied health	12 (00.02)
Main diagnosis	
General exam/routine check-ups	34,050 (64.72)
Diabetes mellitus and glycaemic disorders	6,647 (12.63)
Hypertension and cardiovascular conditions	3,119 (05.93)
Preventive care and follow-ups	2,248 (04.34)
Upper respiratory tract infections	1,712 (03.25)
Genitourinary conditions	1,282 (02.44)
Gastrointestinal conditions	1,204 (02.29)
Musculoskeletal and pain disorders	794 (01.51)
Other metabolic and endocrine disorders	501 (00.95)
Neurological conditions	360 (00.68)
Skin and dermatological conditions	274 (00.52)
Mental health and behavioural disorders	205 (00.39)
Lower respiratory tract conditions	179 (00.34)
Cycle time (min) (median, IQR)	20 (20–21)
Season	
Winter	13,611 (25.87)
Spring	9,141 (17.37)
Summer	13,678 (26.00)
Autumn	16,181 (30.76)

### Multivariable linear regression of factors and cycle time

**Figure 1:**
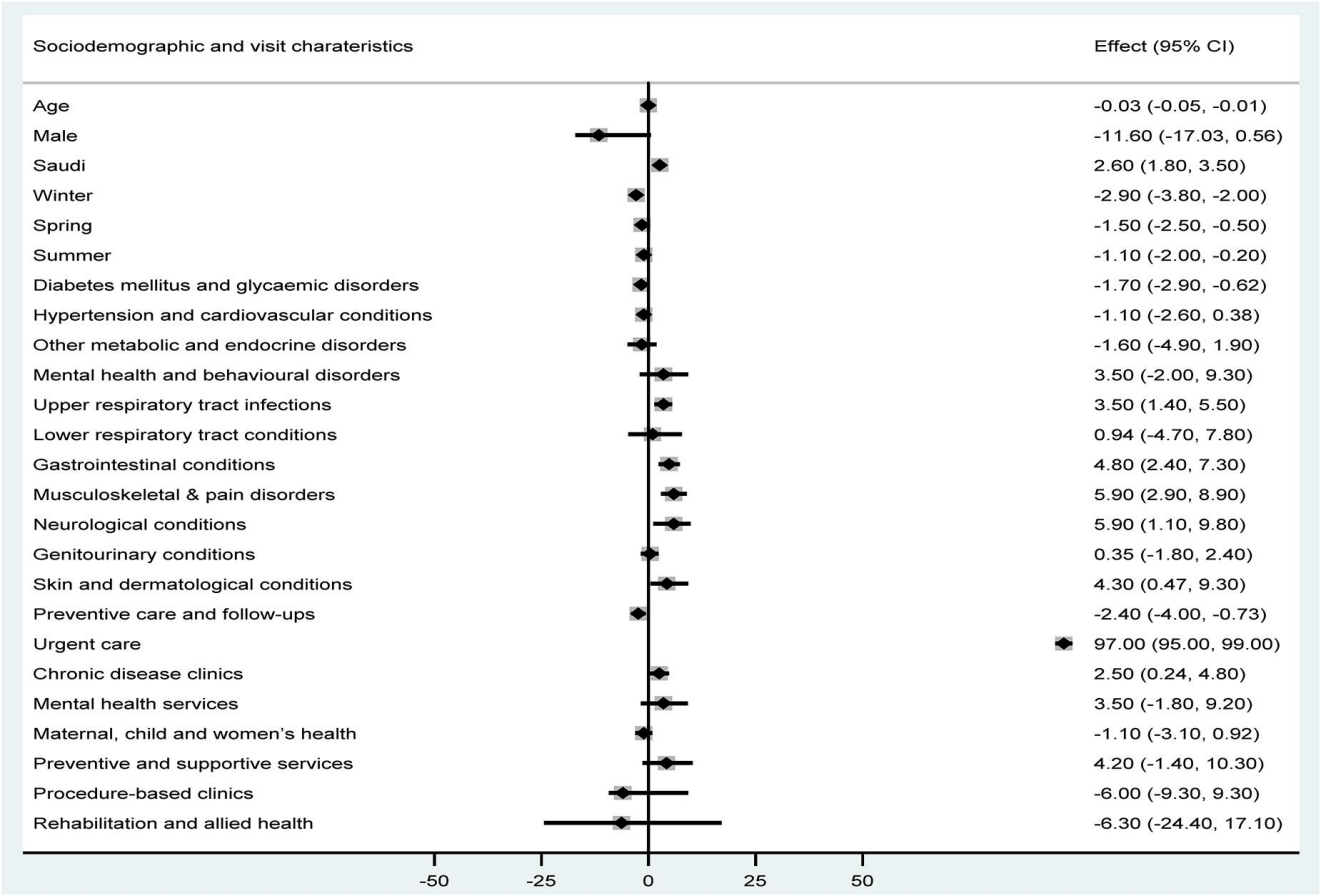
Linear regression coefficients of cycle time.

The multivariable regression results are presented in Figure 1, which illustrates the direction and magnitude of associations between study variables and cycle time. The figure highlights that urgent care was the strongest positive predictor of longer cycle time, while preventive care and diabetes-related visits were associated with shorter cycle time. Age remained a significant predictor after adjustment for all other variables in the model (% change = −0.03, 95% CI = −0.05 to −0.01). Sex was not a significant predictor of cycle time. Nationality was positively associated with cycle time, with visits by Saudi nationals having a significantly longer duration (% change = 2.60, 95% CI = 1.80–3.50). With regard to the season of visit, compared with autumn, all other seasons were associated with significantly shorter cycle times. Compared with visits for “general examinations,” visits with a diagnosis of diabetes and other glycaemic disorders, as well as preventive care and follow-up, were associated with significantly reduced cycle times (% change = −1.70, 95% CI = −2.90 to −0.62, and % change = −2.40, 95% CI = −4.0 to −0.73, respectively). In contrast, compared with general examinations, visits with a recorded diagnosis of upper respiratory tract infections, gastrointestinal conditions, musculoskeletal and pain disorders, neurological disorders, skin and dermatological conditions were associated with significantly increased cycle times. With regard to the types of clinics, urgent care clinics were associated with markedly increased cycle times (% change = 0.97, 95% CI = 95.0–99.0), followed by chronic disease clinics (% change = 2.50, 95% CI = 0.24–4.80), when compared with general family medicine clinics.

### Pattern of visits to the family and community medicine centre

**Figure 2:**
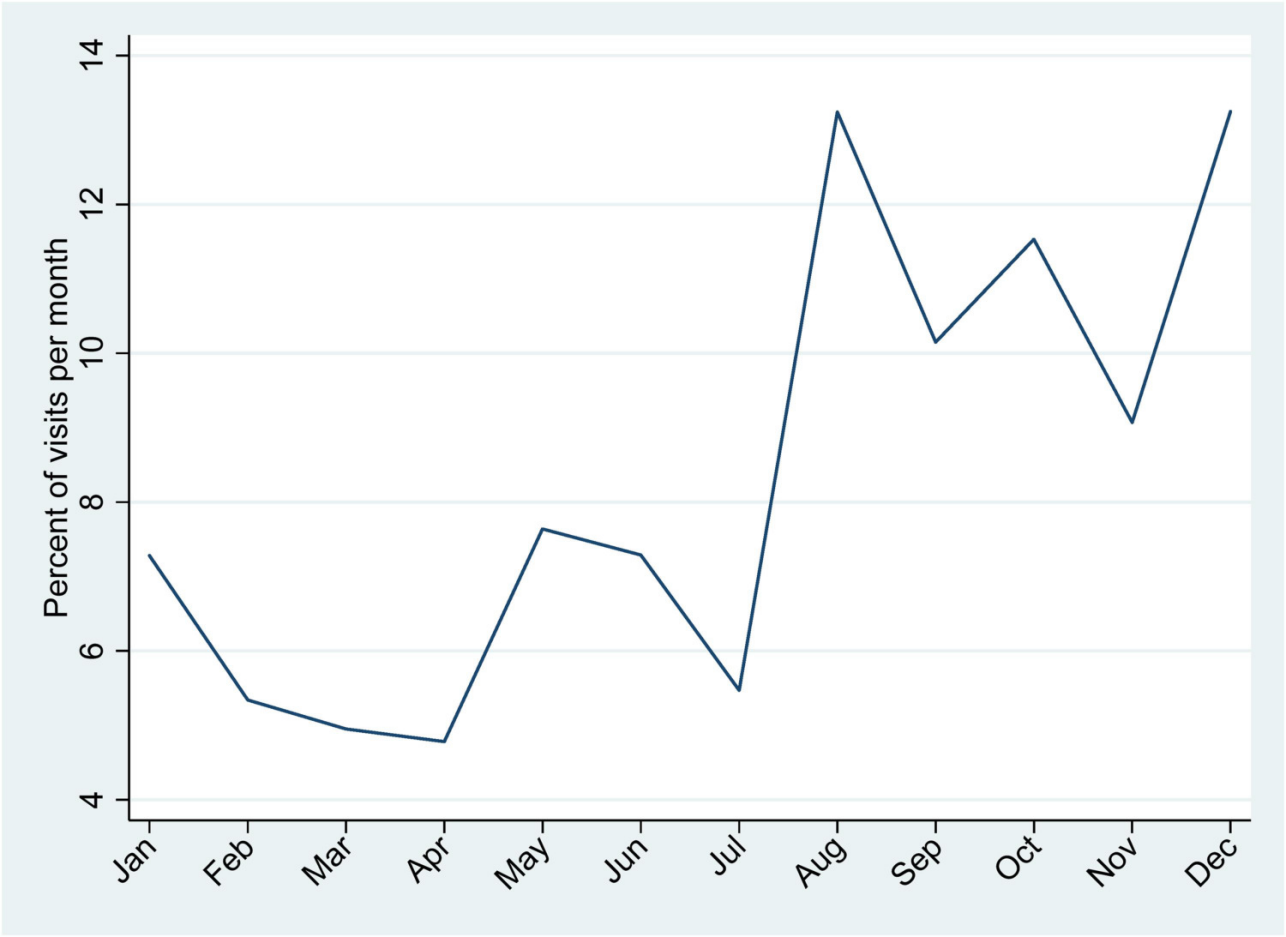
Pattern of visits across months.

As shown in Figure 2, visit volumes increased markedly from August onward, with higher attendance sustained through the later months of the year. This pattern supports the observed seasonal variation in cycle time and suggests that periods of increased demand may have contributed to longer consultation pathway durations. The proportion of visits varied throughout the year, with lower levels observed during the first half, followed by a marked increase in the latter months. Visit proportions declined from January to April, reaching their lowest level in April. An increase was observed in May and June, followed by a transient decline in July. Although a modest decline was noted in September, visit levels remained higher than those observed earlier in the year. Subsequent fluctuations were observed in October and November, with a further increase in December, which recorded one of the highest monthly visit proportions.

## Discussion

This study evaluated patient cycle time in a large academic primary healthcare centre using electronic health record data from 52,611 visits. The median cycle time was 20 min (IQR 20–21), indicating a relatively short duration within the measured clinical consultation pathway. Cycle time is a useful operational measure of the clinical consultation pathway and may help describe variations in visit flow across different clinical contexts (10–12).

Cycle time varied significantly by clinic type, diagnosis, season, and patient characteristics. Urgent care and chronic disease clinics were associated with longer cycle times, whereas preventive and diabetes-related visits were associated with shorter durations. Acute conditions, including upper respiratory, gastrointestinal, musculoskeletal, neurological, and dermatological presentations, were also associated with longer consultation times. In addition, Saudi nationality and autumn visits were associated with increased cycle times, while increasing age was associated with shorter consultation durations.

Although previous studies have extensively examined patient waiting times, evidence specifically addressing patient cycle time remains limited. Reported waiting times vary across healthcare systems, with averages of approximately 30 min in primary care settings in the United Kingdom and substantially longer durations reported in some low-resource settings (15, 16).

Within Saudi Arabia, reported times also vary depending on the healthcare setting and service structure, with some studies reporting averages close to 25 min, while others report longer durations. These variations likely reflect differences in healthcare organization, patient load, and resource allocation (13, 14).

In contrast to several international studies in which chronic disease consultations are associated with longer visit durations (17, 18), this study observed shorter cycle times for diabetes and preventive visits. This may reflect structured care pathways and continuity-based models within academic primary care settings.

Although the observed differences were modest in absolute terms, they may still be relevant in high-volume settings, where small changes can accumulate across large numbers of visits.

The predominance of routine and follow-up visits likely contributed to the shorter overall cycle time observed, reflecting structured workflows and continuity of care within the academic setting.

Longer cycle times observed in urgent care clinics are likely attributable to the unscheduled nature of visits, diagnostic uncertainty, and the need for comprehensive clinical assessment. This finding is consistent with existing literature describing increased consultation durations in urgent care settings (19).

Diagnosis-related variation further supports this interpretation. Acute conditions often require more detailed history-taking, examination, and potential investigations, contributing to longer consultation times. In contrast, chronic disease and preventive visits may benefit from standardized management pathways and patient–physician relationships, resulting in more streamlined consultations.

Cycle time was shorter for chronic conditions such as diabetes and for preventive visits, which may reflect the presence of structured care protocols and continuity of care. These findings differ from some international reports, which show that chronic disease consultations are associated with longer durations (20, 21).

Sociodemographic differences were also observed. Saudi nationality was associated with longer cycle times, which may reflect differences in communication dynamics during consultations. Increasing age was associated with shorter cycle times, a finding that contrasts with some international studies and may reflect familiarity within this care setting (22, 23).

Seasonal variation influenced cycle time, with higher values observed during autumn and winter. This likely reflects increased demand related to the academic calendar and seasonal illnesses, such as influenza, which place additional pressure on clinic capacity (24). These findings highlight the importance of interpreting cycle time within its operational definition and ensuring that quality improvement initiatives are appropriately targeted within the clinical consultation pathway.

### Study strengths and limitations

A key strength of this study is the large, comprehensive dataset derived from electronic health records, enabling robust analysis of cycle time across a wide range of clinical encounters.

One important limitation of this study relates to the structure of the electronic health record system used for data extraction. The available timestamps allowed measurement of total patient cycle time from check-in to physician checkout but did not permit differentiation between specific components of the visit, such as waiting time before consultation, physician consultation time, or time spent on diagnostic procedures. Therefore, the reported cycle time reflects the overall clinical encounter pathway rather than its individual operational components. Previous studies have, in some cases, analysed these elements separately to better understand workflow bottlenecks. Future studies incorporating more granular timestamp data may help further distinguish these components and provide deeper insight into specific contributors to patient flow efficiency. Future research incorporating patient-reported satisfaction measures alongside operational metrics such as cycle time would provide a more comprehensive assessment of healthcare service quality.

## Conclusion

This study found that patient cycle time at an academic primary healthcare centre in Saudi Arabia was relatively short within the measured clinical consultation pathway. However, cycle time varied by clinic type, diagnosis category, season, and patient characteristics. Urgent care clinics and acute presentations were associated with longer cycle times, particularly during periods of increased service demand. These findings highlight areas where operational adjustments, such as workflow optimization and targeted staffing strategies, may support improved management of the clinical consultation pathway in primary healthcare settings.

### Implications for family physicians and family medicine

From an operational perspective, the findings highlight several actionable areas. Urgent care services, which were associated with the longest cycle times, may benefit from dedicated staffing models, rapid triage protocols, and the streaming of minor cases to reduce bottlenecks. Seasonal variation, particularly increased demand during autumn and winter, underscores the need for dynamic workforce planning and flexible clinic scheduling aligned with predictable demand surges.

Acute presentations, which consistently required longer consultation times, may benefit from adjusted appointment durations or clustering within specific clinic sessions to better manage workflow. In contrast, preventive and chronic disease visits, which demonstrated shorter cycle times, could be optimized through standardized care pathways and nurse-led pre-consultation processes.

These targeted strategies may enhance efficiency while maintaining the quality of care within primary healthcare settings.

## Data Availability

The raw data supporting the conclusions of this article will be made available by the authors, without undue reservation.
